# Associations between maternal prepregnancy body mass index and maternal and cord blood metabolome

**DOI:** 10.1210/jendso/bvag035

**Published:** 2026-02-24

**Authors:** Heidi Sormunen-Harju, Polina V Girchenko, Eero Kajantie, Pia M Villa, Esa K Hämäläinen, Emilia Huvinen, Marius Lahti-Pulkkinen, Hannele Laivuori, Katri Räikkönen, Saila B Koivusalo

**Affiliations:** Teratology Information Service, Emergency Medicine, Department of Prehospital Emergency Care, Helsinki University Hospital and University of Helsinki, Helsinki FI-00029, Finland; Department of Obstetrics and Gynecology, Helsinki University Hospital and University of Helsinki, Helsinki FI-00029, Finland; Clinical Medicine Research Unit, MRC Oulu, University of Oulu, Oulu FI-90014, Finland; Department of Psychology and Logopedics, Faculty of Medicine, University of Helsinki, Helsinki FI-00029, Finland; Clinical Medicine Research Unit, MRC Oulu, Oulu University Hospital and University of Oulu, Oulu FI-90014, Finland; Population Health Unit, Finnish Institute for Health and Welfare, Helsinki and Oulu FI-00271, Finland; Department of Clinical and Molecular Medicine, Norwegian University of Science and Technology, Trondheim 7034, Norway; Children's Hospital, Helsinki University Hospital and University of Helsinki, Helsinki FI-00029, Finland; Department of Obstetrics and Gynecology, Helsinki University Hospital and University of Helsinki, Helsinki FI-00029, Finland; Department of Clinical Chemistry, University of Eastern Finland, Kuopio FI-70210, Finland; Department of Obstetrics and Gynecology, Helsinki University Hospital and University of Helsinki, Helsinki FI-00029, Finland; Department of Psychology and Logopedics, Faculty of Medicine, University of Helsinki, Helsinki FI-00029, Finland; Department of Healthcare and Social Welfare, Finnish National Institute for Health and Welfare, Helsinki FI-00271, Finland; Department of Psychology and Logopedics, University of Edinburgh, Edinburgh EH8 9YL, UK; Medical and Clinical Genetics, Helsinki University Hospital and University of Helsinki, Helsinki FI-00029, Finland; Institute for Molecular Medicine Finland, Helsinki Institute of Life Science, University of Helsinki, Helsinki FI-00029, Finland; Department of Obstetrics and Gynecology, Tampere University Hospital, The Wellbeing Services County of Pirkanmaa, Tampere FI-33520, Finland; Center for Child, Adolescent, and Maternal Health Research, Faculty of Medicine and Health Technology, Tampere University, Tampere FI-33100, Finland; Department of Obstetrics and Gynecology, Helsinki University Hospital and University of Helsinki, Helsinki FI-00029, Finland; Department of Psychology and Logopedics, Faculty of Medicine, University of Helsinki, Helsinki FI-00029, Finland; Department of Obstetrics and Gynecology, Helsinki University Hospital and University of Helsinki, Helsinki FI-00029, Finland

**Keywords:** infant, newborn, pregnancy, fetal blood, body mass index, metabolome, obesity

## Abstract

**Context:**

Prepregnancy body mass index (pBMI) is associated with the maternal metabolome during pregnancy. However, evidence remains inconclusive whether pBMI is also associated with alterations in the fetal cord blood metabolome, and whether pBMI modifies the associations between maternal and fetal metabolomes.

**Objective:**

This work aimed to examine whether pBMI is associated with the fetal cord blood metabolome and whether maternal and fetal metabolomes are associated and vary according to the degree of maternal pBMI.

**Methods:**

We derived pBMI from medical records and tested it in relation to 95 cord blood metabolic measures of 1702 newborns in the PREDO, RADIEL, and ITU studies. We tested the associations between maternal and fetal metabolomes and moderation by pBMI in 556 mother-child dyads of the PREDO and RADIEL studies contributing maternal blood samples at 3 time points during pregnancy.

**Results:**

In the meta-analysis of the 3 studies, higher pBMI was associated with 12 of the 95 cord blood metabolic measures, including lower levels of high-density lipoprotein–associated measures and higher levels of branched-chain and aromatic amino acids, as well as ketone bodies. Associations between maternal and fetal metabolomes were significant for 61 of the 95 measures; 26 of the 95 associations were modified by maternal pBMI, being stronger among mothers with obesity than those without.

**Conclusion:**

Maternal pBMI is associated with alterations in fetal cord blood metabolome. Maternal and fetal metabolomes are associated, and associations vary according to maternal pBMI.

The obesity (body mass index, BMI ≥ 30) rate continues to climb globally: By 2030, one-fifth of all women are predicted to be affected [1]. The prevalence of maternal prepregnancy obesity is climbing accordingly [2]. In addition to increasing the risk for various short-term pregnancy complications, maternal prepregnancy obesity is associated with an increased risk for several long-term adverse outcomes in the offspring, including obesity [3, 4], insulin resistance [5, 6], cardiovascular diseases [7, 8], and mental and behavioral disorders [9].

The mechanisms mediating the adverse effects of obesity on the offspring are probably multifactorial, but perturbations in the maternal metabolome seem to play a role [10-12]. Indeed, we and others have demonstrated that obesity is associated with widespread alterations in the maternal metabolomic profile during pregnancy, including perturbations in lipoprotein-related measures, triglycerides, some amino acids (AAs), fatty acids (FAs), and inflammation markers [13-15].

However, the evidence remains inconclusive whether maternal obesity and related alterations in the maternal metabolome during pregnancy are associated with the fetal cord blood metabolomic profiles. The fetal cord blood metabolome is a combination of metabolites originating from the maternal side and crossing the placenta, and those metabolized and synthesized by the placenta and fetus. To the best of our knowledge, of the 7 studies testing associations between maternal prepregnancy BMI (pBMI) and fetal cord blood metabolic profiles, 4 have been small scale with sample sizes varying from 24 to 116 [16-19]. In the 3 larger-scale studies with the number of mother-child dyads between 321 and 1600 [20-22], pBMI has been associated with no cord blood measures [20] or with several measures including branched-chain amino acids (BCAAs), aromatic amino acids (AAAs), and carnitines [21]. The disagreement in findings may be related to the differences in sample sizes, population characteristics, and differences in the metabolic panels, and also to whether maternal pBMI was measured or self-reported, which is well known to carry a bias [23, 24]. Moreover, while these and other studies have reported modest-to-moderate associations between maternal and fetal cord blood metabolomes [17, 21, 22, 25], we are not aware of studies testing whether these associations vary by maternal pBMI. As obesity is becoming more common among women of reproductive age, it is crucial to gain deeper insights into the associations between maternal and fetal metabolomes.

Thus, we measured fetal cord blood metabolome with a widely used nuclear magnetic resonance (NMR) platform. We tested the associations between maternal pBMI derived from medical registries and 95 fetal cord blood metabolic measures in a meta-analysis of 3 independent Finnish studies including altogether 1702 mother-child dyads. We then examined whether maternal and respective fetal cord blood metabolic measures were associated, whether these associations varied based on gestational timing of maternal measurements, and whether these associations were moderated by maternal pBMI. We addressed the latter questions in 556 mother-child dyads in 2 of the studies, both of which provided 3 maternal pregnancy blood samples.

## Materials and methods

### Participants

Our study population came from 3 Finnish studies, the Prediction and Prevention of Pre-eclampsia and Intrauterine Growth Restriction (PREDO) study [26], the Finnish Gestational Diabetes Prevention (RADIEL) study [27], and the InTraUterine Sampling in Early Pregnancy Study (ITU) [28]. Supplementary Fig. S1 presents the flowchart of the study [29].

The PREDO study recruited between 12 and 14 gestational weeks (GWs) 1079 pregnant women with known risk factor status for preeclampsia and intrauterine growth restriction from 10 hospitals [26]. The risk factors included preeclampsia, gestational diabetes, intrauterine growth restriction or fetal demise in the previous pregnancy, prepregnancy obesity, chronic hypertension, type 1 diabetes, maternal age younger than 20 or older than 40 years, systemic lupus erythematosus, and Sjögren syndrome. Of all women, 969 had at least 1 and 110 had none of these risk factors. A subgroup with a second-degree diastolic notch in the uterine blood flow were randomly assigned to receive low-dose aspirin (n = 61) or placebo (n = 60) for preventing preeclampsia. This sample was appended by 75 women regardless of risk factor status, who donated placenta and fetal cord blood samples, totaling 1154 pregnancies. From these 1154 pregnancies, 923 (80.0%) women provided umbilical cord blood samples for metabolomic analyses. Of these 923 pregnancies, maternal blood samples were available from 341 women at 3 time points during pregnancy at a median 12.6 (interquartile range [IQR] 11.6-13.4), 19.3 (19.0-19.7), and 27.0 (26.6-27.6) GWs.

The RADIEL study recruited 720 women who were planning a pregnancy or in the first half of pregnancy (before 20 GW) into a randomized clinical trial to prevent gestational diabetes by lifestyle intervention among high-risk women (prior gestational diabetes and/or prepregnancy obesity). Of these women, 365 were randomly assigned to the intervention group receiving advice on diet and physical activity, and 355 to the control group treated with standard care. Of these 720 women, 611 conceived with a singleton pregnancy and umbilical cord blood samples were obtained at delivery from 369 newborns. Of these 369 pregnancies, maternal blood samples were available from 215 women at 3 time points during pregnancy at a median 13.0 (IQR 11.9-14.3), 23.1 (22.6-24.1), and 35.1 (34.4-35.7) GW.

The ITU study was designed to assess biological mechanisms in the programming of health and disease after exposure to prenatal adversities and recruited, between 9 and 22 GWs, 943 women taking part in the national voluntary prenatal testing for fetal chromosomal abnormalities. Of these women, 544 had a positive screening result but were cleared for fetal chromosomal abnormalities; 399 had a negative screening result. In these 943 pregnancies, umbilical cord blood samples were obtained at delivery from 410 newborns.

A total of 1702 mother-child dyads from these 3 studies comprised the analytic sample to study the associations between maternal pBMI and fetal cord blood metabolome. In comparison to the dyads without cord blood sample in these 3 studies (n = 931), mothers in the dyads with cord blood sample were younger (33.4 vs 34.0 years; *P* = .008), the birth weight was higher (3578 vs 3498 g; *P* = .006), and gestational age at delivery was longer (39.9 vs 39.5 GW; *P* < .001). The analytic sample to study associations between maternal and fetal metabolome and variation according to maternal pBMI comprised 556 mother-child dyads from the PREDO and RADIEL studies. There were no differences in the background characteristics in PREDO-RADIEL between those 556 dyads with both cord blood and maternal samples available, and those 1134 dyads missing either maternal, cord blood, or both samples.

All study participants provided informed consent, and the study protocols were approved by the ethics committee of the Helsinki and Uusimaa Hospital District.

### Maternal prepregnancy body mass index

In all 3 cohorts, the Finnish Medical Birth Register provided data on maternal prepregnancy weight and height verified at the first visit to the antenatal clinics at 7 to 10 GWs. Prepregnancy weight and height measured at the last study visit before pregnancy was used for the participants recruited before pregnancy in the RADIEL study. In accordance with World Health Organization guidelines, obesity was defined as pBMI greater than or equal to 30 and nonobesity as pBMI less than 30 [30].

### Maternal and fetal cord blood metabolomic profiling

In all 3 cohorts, maternal venous blood samples were drawn from the antecubital vein between 7 and 10 Am after at least a 10-hour overnight fast, and the cord blood sample was extracted immediately after the child was born. In PREDO and ITU, plasma, and in RADIEL, serum was separated immediately and stored at −80 °C until analysis. A high-throughput proton NMR metabolomics platform quantified 225 metabolic measures in maternal samples and 110 in fetal cord blood samples using the Nightingale Health Quantification Library 2020 (Nightingale Health Ltd). The platform and its applications have been described in detail previously [31]. In brief, the panel includes the metabolomic analysis of lipid, lipoprotein, and glucose metabolism, AAs, FAs, ketone bodies, and a biomarker of low-grade inflammation and has been used in numerous studies of pregnant and nonpregnant populations [32-34]. Based on earlier studies using the same metabolomic platform in pregnant and general populations of adults, 95 measures were considered relevant in comprising an adequate picture of the metabolism, and these were selected as the primary outcomes. The measures not included were compositions of various lipoprotein subclasses and relative lipoprotein lipid concentrations. Due to the high level of intraclass correlation of the metabolic measures across the 3 measurement points (*R* = 0.56-0.89; *P* < .001) and to our previous results showing pregnancies with obesity to be characterized by persistent metabolic perturbations throughout pregnancy [15], we used the mean of the 3 maternal measurements across pregnancy in the analyses.

### Covariates and confounders

Based on the literature, maternal age in years [14], parity (primiparous vs multiparous) [32], smoking or alcohol use (yes vs no) at any time during pregnancy [32], education (tertiary vs basic/secondary) [13], and child sex were considered potential confounders and were adjusted for in the models examining the effects of pBMI on cord blood metabolic measures, and the effects of maternal metabolic measures on cord blood metabolic measures. Education and alcohol use were reported in early pregnancy; all other data were derived from the Finnish Medical Birth Register. We also made adjustments for child's gestational age and birth weight. However, as pBMI is known to be associated with the child's gestational age and birth weight, which are, in turn, associated with cord blood metabolic measures, gestational age and birth weight may mediate, rather than confound, the associations between pBMI and the cord blood metabolic measures. Cohort was considered a covariate in all analyses.

### Statistical analysis

We log-transformed the metabolic measures to normalize their distributions and analyzed the values in cohort-specific standardized units. To examine the association between maternal pBMI and cord blood metabolic measures, we applied a one-stage individual participant data meta-analytic approach combining the harmonized data pooled across the PREDO, RADIEL and ITU studies. We conducted individual participant data meta-analysis with linear mixed-effects models with the cohort as a random effect to allow for differences in clustering of the participants between the 3 cohorts. As effect size we report the estimates, which represent change in the metabolic measure in SD units per 1-unit change in maternal pBMI. As the metabolic measures are highly correlated, the Bonferroni correction for multiple testing may be overly conservative and raise the risk of type II error [35]. To reduce this risk, principal components analysis has been applied as a multiple testing correction method to identify the effective number of independent tests. We identified 34 principal components explaining 99% of the variation in the 95 cord blood metabolic measures used as the primary outcomes. Thus, in these analyses a 2-sided *P* value less than .00147 (0.05/34) translating into 99.9% CIs was used to infer statistical significance.

We examined the associations between maternal and respective cord blood metabolic measures in a combined PREDO-RADIEL sample using generalized linear regression. To examine whether the associations between maternal and cord blood metabolic measures varied based on gestational timing of the maternal metabolic measures, we used reverse temporal mixed-effects regression analyses. In these analyses, the repeated maternal metabolic measures represented the within-person outcome variables, and GW at blood sampling represented the within-person predictor and fetal cord blood metabolic measures as the between-person predictor variables, and their interaction tested whether the associations varied based on gestational timing of the maternal blood sampling. This analysis thus tested whether the associations between maternal and cord blood metabolic measures changed as gestational age progressed. We defined unstructured covariance and first-order autoregressive error covariance matrices, and specified a person-specific random intercept and time-varying slopes. As each exposure (maternal metabolic measure) was tested for one outcome (respective cord blood metabolic measure), *P* value less than .05 was used to infer statistical significance in these analyses. As effect size we report estimates representing change in the cord blood metabolic measure in SD units per each SD unit change in the corresponding maternal metabolic measure, and change in the strength of these associations per each SD unit change in gestational age, and their 95% CIs.

We then tested whether the associations between maternal and respective cord blood metabolic measures in the combined PREDO-RADIEL study differed according to the level of maternal pBMI by adding the interaction term maternal pBMI × maternal metabolic measure to the models in addition to their main effects. Because we used pBMI as a continuous variable in the interaction analyses, we conducted subanalyses for display purposes to facilitate interpretation for all interaction terms with a *P* value less than .05. In these subanalyses we tested associations between maternal and cord blood metabolic measures separately among mothers with obesity (≥30) and nonobesity (<30).

We present the associations as adjusted for cohort, maternal age, education, parity, substance use during pregnancy, and child sex, and further for child's gestational age and birth weight. No missing values were imputed since mixed models allow missing data. If the measure was below the detection limit, we used the nonzero minimum value of that measure multiplied by 0.9.

Statistical analyses were performed using SAS 9.4 (SAS Institute Inc).

## Results

Table 1 presents by cohort the characteristics of the 1702 mother-child dyads that provided data for studying associations between maternal pBMI and fetal cord blood metabolic measures. Supplementary Table S1 [29] presents the characteristics of those mother-child dyads who, in addition to cord blood metabolomic data, provided maternal metabolomic data to study the associations between these two, and moderation by maternal pBMI.

**Table 1:** Background characteristics of the 1702 mother-child dyads by cohort

Characteristics	PREDO (N = 923)	RADIEL (N = 369)	ITU (N = 410)	TOTAL (N = 1702)
**Maternal**				
Age, mean (SD), y	33.1 (5.6)	32.9 (4.4)	34.5 (4.9)	33.4 (5.2)
BMI, mean (SD)	27.1 (6.4)	31.5 (5.9)	23.7 (4.0)	27.2 (5.7)
Obesity, n (%)	312 (34%)	243 (66%)	28 (6.8%)	583 (34%)
Primiparous, n (%)	291 (32%)	121 (33%)	233 (57%)	645 (38%)
Education level, n (%)				
Secondary or lower	404 (44%)	256 (69%)	72 (18%)	732 (43%)
Tertiary	496 (54%)	112 (30%)	328 (80%)	936 (55%)
Data not available	23 (2.5%)	1 (0.3%)	10 (2.4%)	34 (2%)
Smoking or alcohol use during pregnancy, n (%)	162 (18%)	28 (7.6%)	17 (4.1%)	207 (12%)
Data not available	111 (12%)	4 (1.1%)	0	115 (6.8%)
**Offspring**				
Birth weight, mean (SD), g	3551 (550)	3706 (517)	3523 (481)	3578 (526)
Gestational age at delivery, mean (SD)	39.8 (1.6)	39.9 (1.6)	40.0 (1.5)	39.9 (1.6)
Sex, female, n (%)	441 (47.8%)	175 (47.4%)	203 (49.5%)	819 (48%)

No missing data unless stated otherwise.

Abbreviations: BMI, body mass index; ITU, InTraUterine Sampling in Early Pregnancy Study; PREDO, Prediction and Prevention of Pre-eclampsia and Intrauterine Growth Restriction study; RADIEL, Finnish Gestational Diabetes Prevention study.

### Association between maternal prepregnancy body mass index and cord blood metabolome

**Figure 1:**
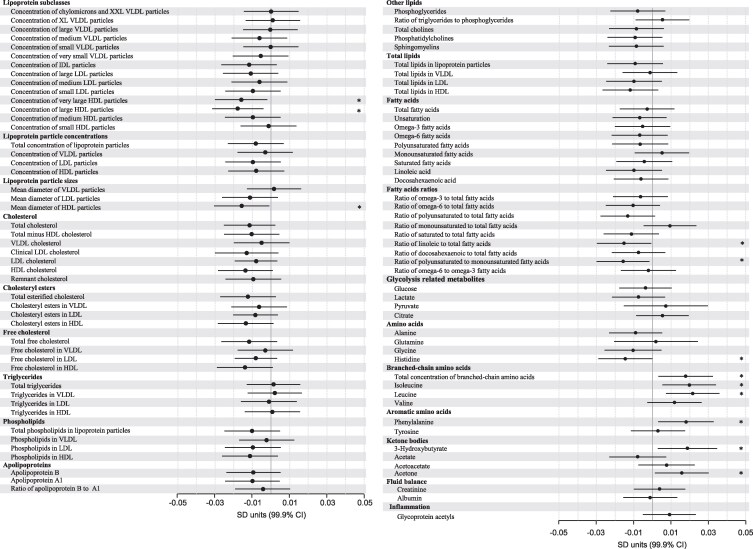
Association between maternal prepregnancy body mass index (pBMI) and cord blood metabolic measures in the meta-analysis of 3 studies (PREDO, RADIEL, and ITU) with 1702 mother-child dyads. Dots represent mean change (increase or decrease) in cord blood metabolic measures in SD units per 1-point increase in maternal pBMI, and bars their 99.9% CIs. The model has been adjusted for cohort, maternal age, education, parity, substance use during pregnancy, and offspring sex. Statistically significant associations are marked with an asterisk.

In the meta-analysis of all 3 studies, in the models adjusted for cohort, maternal age, education, parity, substance use during pregnancy, and child sex, maternal pBMI was significantly associated with levels of 12 metabolic measures (Fig. 1). Higher maternal pBMI was associated with smaller mean diameter of high-density lipoprotein (HDL) particles, lower concentration of large and very large HDL particles, lower ratios of linoleic acid (LA) to total FAs, and of polyunsaturated (PUFA) to monounsaturated (MUFA) FAs, lower level of histidine, and higher levels of all BCAAs, except for valine, and higher levels of phenylalanine and ketone bodies, that is, 3-hydroxybutyrate and acetone. In the models adjusted for the child's gestational age and birth weight, pBMI remained statistically significantly associated with lower concentration of large HDL particles and lower ratio of PUFAs to MUFAs (Supplementary Fig. S2) [29].

### Association between maternal and cord blood metabolic measures

**Figure 2:**
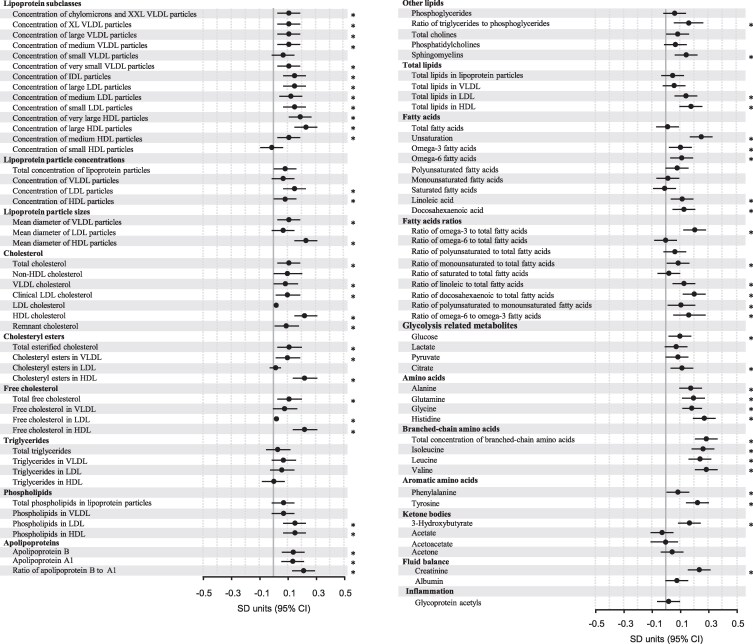
Association between maternal and cord blood metabolic measures in PREDO-RADIEL (n = 556). Dots represent change in cord blood metabolic measures in SD units per 1-SD unit change in maternal measure, and bars their 95% CIs. The model has been adjusted for cohort, gestational age at blood sampling, maternal age, education, parity, substance use during pregnancy, and offspring sex. Statistically significant associations are marked with an asterisk.

In the PREDO-RADIEL study, in the model adjusted for cohort, maternal age, education, parity, substance use during pregnancy, and child sex, we found statistically significant associations between maternal metabolic measures averaged across the 3 gestational sampling points and respective cord blood metabolic measures in 61 of the 95 metabolic measures (Fig. 2). Associations were found in all different classes of metabolic measures, including lipoproteins, lipids, FAs, and AAs, but no association was found between maternal and cord blood triglycerides.

Analyses testing whether the associations between maternal and cord blood metabolic measures varied based on gestational timing of the maternal measures showed that in 5 metabolic measures the maternal and fetal associations became stronger as the pregnancy progressed (increase in the strength of the associations per 1-week increases in GW: 0.002-0.005 SDs). These metabolic measures included 4 lipids (concentration of large low-density lipoprotein [LDL] particles, concentration of LDL particles, average diameter of very-low-density lipoprotein particles, and apolipoprotein B) and one AA (alanine) (Supplementary Table S2) [29].

### Do associations between maternal and cord blood metabolic measures vary by the level of maternal prepregnancy body mass index?

**Figure 3:**
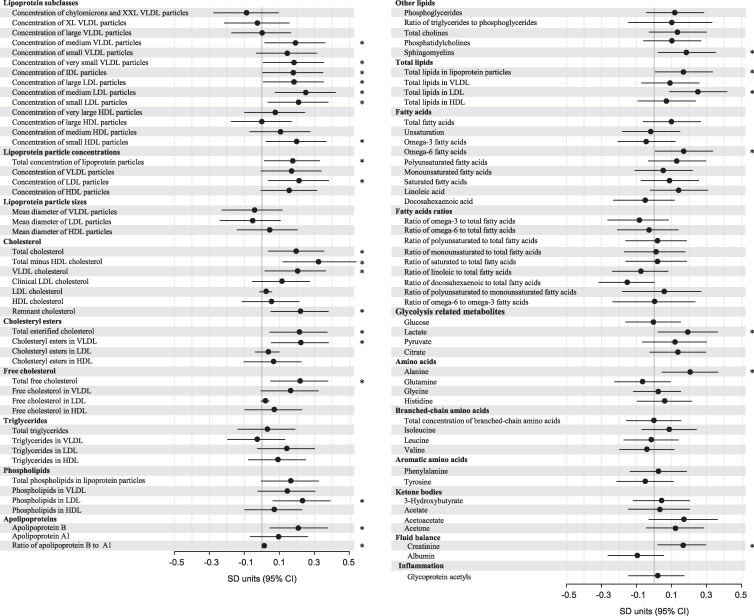
Interaction of maternal metabolic measures with maternal prepregnancy body mass index on cord blood metabolic measures in PREDO-RADIEL (n = 556). Dots represent mean interactions, and bars their 95% CIs. The model has been adjusted for cohort, maternal age, education, parity, substance use during pregnancy, and offspring sex. Statistically significant interactions are marked with an asterisk.

**Figure 4:**
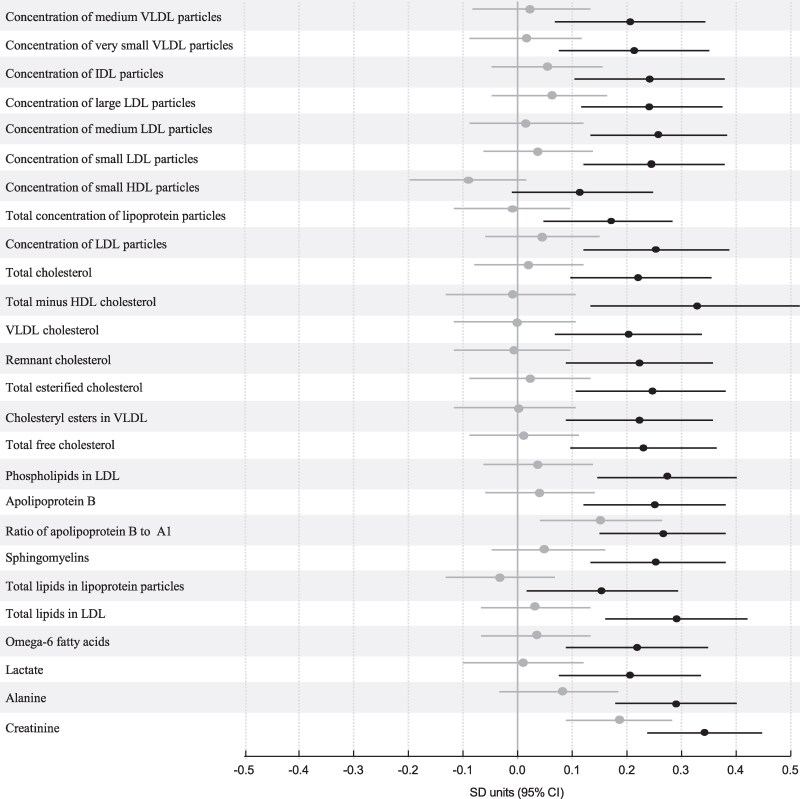
Association between maternal and cord blood metabolic measures in PREDO-RADIEL in women with nonobesity (n = 308; gray lines) or with obesity (n = 248; black lines). Dots represent mean increase or decrease of cord blood measures in SD units per 1-SD unit increase in maternal measure and bars their 95% CIs. The model has been adjusted for cohort, maternal age, education, parity, substance use during pregnancy, and offspring sex.

In the PREDO-RADIEL study, 26 of the 95 tested interactions were statistically significant (Fig. 3); associations between maternal and respective fetal cord blood levels of various sizes of very-low-density lipoprotein, intermediate-density lipoprotein, and LDL particles, and small HDL particles, many cholesterol-related measures, apolipoprotein B and its ratio to apolipoprotein A1, ω-6 FAs, lactate, alanine, and creatinine varied significantly according to the level of maternal pBMI. Subgroup analyses showed that all these 26 associations were stronger in mothers with obesity compared to those without (Fig. 4).

## Discussion

In this meta-analysis of 3 studies, we found that higher maternal pBMI was associated with lower levels of several HDL-related measures and some FA ratios, and with higher levels of several AAs and ketone bodies in fetal cord blood. We also found in 2 of the studies that, of 95 maternal metabolic measures averaged across the 3 time points in early, mid, and late pregnancy, 61 were associated with the respective metabolic measures in the fetal cord blood. The associated metabolic measures included many lipids, FAs, and AAs. We demonstrated that, except for 5 metabolic measures, 4 lipids and 1 AA, the associations between maternal and fetal metabolic measures remained stable from early to late pregnancy. Furthermore, in the subsample combining 2 cohorts with 3 maternal pregnancy samples, the associations between maternal and respective fetal cord blood levels were moderated by maternal pBMI. The associations between maternal and fetal metabolic measures were stronger among mothers with obesity than among mothers with nonobesity. The metabolic measures moderated by maternal pBMI were mainly lipids and lipoproteins.

We found higher pBMI to be associated with several perturbations in the cord blood metabolome: lower levels of the largest HDL particles and smaller size of HDL particles, adverse FA ratios, higher levels of BCAAs, of 1 of the AAAs, that is, phenylalanine, and of 2 ketone bodies, and lower level of histidine. Our finding concerning the association with BCAAs and phenylalanine is supported by 1 of the 3 larger-scale studies assessing the association between pBMI and cord blood measures, namely the ethnically diverse Hyperglycemia and Adverse Pregnancy Outcomes (HAPO) study with 1600 newborns from 4 ancestry groups [21]; the similar association was found in the meta-analysis of the 4 groups, but not in individual ancestry groups. The metabolic assessment in HAPO study included 65 measures, a narrower set than ours. A recent study by Mansell [22] with 782 newborns used the same NMR-based Nightingale method as our study, but the population in their study was according to the self-reported maternal anthropometrics leaner (mean maternal BMI of 25.3) than in our study (27.2). After adjustment for birthweight, Mansell found only 1 of the reported 76 cord blood measures to be associated with higher pBMI, and that was higher triglyceride-to-phosphoglyceride ratio. Similarly in our study, most of the associations were no longer statistically significant after adjustment for birth weight and gestational age. This attenuation in the associations reflects that child gestational age and birth weight may mediate the associations between pBMI and fetal cord blood metabolic profiles. Self-reported maternal weight was used in the Desert study with a total of 321 women [20], but with a very low (n = 19) number of women with obesity; no association was found between maternal BMI and cord blood metabolome. One of the smaller studies with 116 newborns also found the similar positive association between pBMI and higher BCAAs as the HAPO study and ours [16].

As we [15] and others [13, 14] have shown, maternal obesity is associated with higher maternal circulating BCAA and AAA levels, metabolites with a well-established association with insulin resistance in the adult population [36, 37]. We have now demonstrated that maternal obesity is also associated with higher cord blood levels of these AAs. AAs are essential for fetal growth and development and are actively transported across the placenta [38]. Both high and low levels of cord blood BCAAs have been demonstrated to be detrimental; high levels have been associated with intrauterine growth restriction [39] and a greater risk for later development of attention-deficit hyperactivity [40] disorders in the offspring, and low levels with the risk of childhood overweight or obesity [41]. Transport of AAs has been suggested to be regulated to protect the fetus from overnutrition [38], and this regulatory effect may be interfered with by obesity.

Higher pBMI was associated with lower cord blood ratio of PUFAs to MUFAs, PUFAs to total FAs, and LA to total FAs in our study, similarly to the findings of adverse cord blood FA profiles with maternal obesity in a previous smaller study comprising 103 mother-child dyads [42]. Obesity-associated inflammation impairs placental function and alters the storage of FAs; an ex vivo model has demonstrated reduced mobilization of free FAs in the placentas of women with obesity and, thus, lower docosahexaenoic acid levels in the fetal circulation [43]. Some studies have found an association between cord blood FA levels and birth weight or neonatal adiposity [44-46], but there is no evidence of an association between cord FA levels and later offspring adiposity [47]. Elevated cord blood acylcarnitine level, that is, FA metabolites, was associated with the later development of autism spectrum disorder and attention-deficit syndrome in the Barwon Infant Study with more than 1000 mother-child dyads [48]. In the analysis of participants in the UK Biobank, an adult population, circulating FA ratios were biomarkers for numerous diseases and the association with type 2 diabetes was strong [49]. The role of PUFAs at early ages may, however, be different from the role in adults, a speculation supported by a discovered association between relatively lower levels of cord blood docosahexaenoic acid and lower diastolic blood pressure at age 9 years, known to be beneficial for cardiovascular health [50].

Lower cord blood level of the largest HDL particles and smaller size of HDL particles were associated with higher maternal pBMI in our study. Maternal obesity greatly affects the metabolism, composition, and function of maternal HDL [15, 51]. Our findings are consistent with previous studies showing lower levels [51-53] and impaired function [51] of cord blood HDL cholesterol following maternal obesity. Low maternal second- and third-trimester HDL cholesterol measures were associated with offspring early ascending growth [54] in a recent study. Large HDL particles are antiatherogenic, and the inverse association between cardiovascular risk and HDL cholesterol has been well established in adult populations [55, 56]. A recent study found an association between low cord blood level of HDL and children's teacher-rated psychosocial competence at age 5 years [57], but the few studies exploring later childhood growth or adiposity have not indicated any association with cord blood HDL [58, 59].

Our study found a statistically significant association between a great number of metabolic measures in maternal mean pregnancy and cord blood levels at delivery, and measures spanned across all different classes with the exception of triglycerides, similarly to the HAPO study [21]. In previous studies, the degree of association between maternal and cord blood measures has varied according to the panel of metabolic measures in the selected method [16, 18, 21, 60]. A Norwegian Mother, Father and Child Cohort Study with 679 trios [25] and using the same panel as in our study also demonstrated associations between maternal and cord blood levels of several lipids, FAs, and AAs, but similar to our study, no association between maternal and cord blood triglycerides. In our study, associations between maternal and fetal metabolic measures remained stable across gestation with few exceptions. For example, we observed strengthening of the association between maternal and fetal alanine from early to late pregnancy, which is consistent with the active transport of AAs across the placenta. Mechanisms behind the associations between maternal and cord blood levels of the metabolites not transported across the placenta are probably multifactorial. A recent study using the same NMR metabolomics panel in more than 136 000 participants found more than 8000 genetic associations of circulating metabolic measures [61]; genetic similarities may explain some of the associations in the levels of these measures between mother and the offspring. Despite this, the Mother, Father and Child Cohort Study found only one measure with an association between paternal and cord blood levels, ratio of ApoB to ApoA1. The lack of more metabolic associations between the father and the fetus highlights the importance of the shared feto-maternal milieu during pregnancy. The presence of a number of associations between maternal and cord blood metabolomes and the stability of the associations throughout pregnancy points to a critical need for preventive interventions against the adverse effects of maternal obesity before conception.

This is the first study to demonstrate that maternal pBMI modifies the association between maternal metabolic measures in pregnancy and respective cord blood levels at delivery. Placentas of women with obesity are frequently larger, and such placentas exhibit histopathological changes of inflammation and underperfusion [62]. In our study, compared to those with nonobesity, women with obesity had stronger associations between maternal and cord blood levels of several lipoprotein subfractions, apolipoprotein B, ω-6 FAs, lactate, alanine, and creatinine. The modifying effect on many lipid measures may be relevant as lipids play a central role in early and later childhood growth and development [63-65] as well as in risk for metabolic disease [66]. We found the association between maternal and cord blood levels of small atherogenic HDL particles and of a PUFA, ω-6 FA, to be stronger in women with obesity than those without. As already discussed, the implications of early-life circulating FA levels are elusive and further studies are needed. Of the AAs, a modifying effect by pBMI was found only in alanine.

Several biological pathways may lead to a mechanistic connection between the materno-fetal metabolome and later offspring outcomes [67]. These pathways may include the altered expression of placental nutrient transporters, seen in maternal obesity, when the placenta exhibits increased expression of nutrient transporters, such as FA [68] or AA transport proteins [69, 70]. Furthermore, in placentas of mothers with obesity, high lipid levels may induce oxidative stress and impair mitochondrial function [71]. Exposure, during critical windows of development, to abnormal metabolite levels may also lead, in the fetus, to epigenetic changes affecting metabolism-related gene expression [72]. Further studies are warranted to explore the effect of fetal cord blood metabolic alterations on long-term health and developmental outcomes in children as well as the underlying mechanisms.

The strength of our study lies in combining 3 well-documented Finnish study cohorts into a meta-analysis including more than 1700 mother-child dyads. Three maternal blood samples in pregnancy were available only in 2 of these study cohorts, limiting the number of dyads in the analyses of materno-fetal associations, but these 2 cohorts were enriched with a high number of women with obesity. We used a targeted metabolic panel that has been widely used, and many of these measures have been validated against conventional laboratory techniques. Limitations of our study may arise from the original study interventions, but we have earlier shown interventions not to be associated with the maternal metabolome [15]. A potential limitation may also derive from the different samples in the studies, serum and plasma, but bias of different samples seems minimal [73] and SD scaling and adjustment for cohort address this issue. Although BMI correlates strongly with metabolic risk factors and disease outcomes, additional measures, such as maternal anthropometric data (including waist circumference or body composition), or indicators of glucose metabolism or insulin resistance, could have provided a more comprehensive perspective on the study's research questions, but these data were not available. The large population adds power in our study, but the high-resource Nordic setting and eligibility criteria related to risk factors for preeclampsia, intrauterine growth restriction, and gestational diabetes may limit the generalizability of the results.

In conclusion, our large study supports the previous findings of an association between high maternal pBMI and perturbations in cord blood metabolome, and the findings of an association between levels of many metabolic measures in maternal and cord blood. Furthermore, we have presented novel evidence of maternal obesity modifying the association between maternal and cord metabolome. Future studies are needed to explore the influence of these alterations on offspring growth and development as they may help us understand determinants of fetal and infant well-being.

## Data Availability

Datasets generated during the current study are not publicly available but will be made available on reasonable request. Requests are subject to further review by the national registry authority and by the ethics committees.

## Abbreviations

AA: amino acid
AAA: aromatic amino acid
BCAA: branched-chain amino acid
BMI: body mass index
FA: fatty acid
GWs: gestational weeks
HAPO: Hyperglycemia and Adverse Pregnancy Outcomes Study
HDL: high-density lipoprotein
IQR: interquartile range
ITU: InTraUterine Sampling in Early Pregnancy Study
LA: linoleic acid
LDL: low-density lipoprotein
MUFA: monounsaturated fatty acid
NMR: nuclear magnetic resonance
pBMI: prepregnancy body mass index
PREDO: Prediction and Prevention of Pre-eclampsia and Intrauterine Growth Restriction study
PUFA: polyunsaturated fatty acid
RADIEL: Finnish Gestational Diabetes Prevention study
